# Medical practice and placebo response: an inseparable bond?

**DOI:** 10.1007/s00508-020-01626-9

**Published:** 2020-03-24

**Authors:** Sandra Jilch, Ruken Sel, Shahrokh F. Shariat

**Affiliations:** Medical University of Vienna, Vienna, Austria

**Keywords:** Placebo, Medicine, Placebo effect, Nocebo, Placebo history

## Abstract

The history of medicine and the history of placebo are closely intertwined. To understand placebo and its effects this article gives a brief overview about its history, the possible mechanisms of action and its counterpart, nocebo.

The Catholic Church used placebo around the sixteenth century for the separation from real and incorrect exorcisms, but it needed Henry Beecher during World War II to quantify the placebo effect as control arm in well-designed studies.

Until today the different mechanisms of action of placebo remain poorly researched. Understanding them would allow its effect to be modulated to better serve in research and clinical settings. Expectation, psychosocial context and conditioning play a significant role in the effect size and amplitude.

The counterpart, nocebo, is even less investigated, even it is commonly observed as adverse effects during medical treatments.

*Conclusion:* Placebo and nocebo are both underestimated and underresearched in their value. Through further investigation doctors could strengthen the placebo response and prevent adverse effects to help their patients at low cost. These techniques would benefit the patient-doctor relationship, which is the alter of a trust-based successful therapy.

## Introduction

The history of medicine, as we know it today and the history of placebo are closely intertwined, as many treatments from the past relied heavily on the placebo effect, such as bloodletting for fever and other ailments [1]. Until the first half of the twentieth century, placebo was commonly used for the treatment of neurotic or inadequate patients [2].

There are several types of placebo distinguished today as pure and impure placebo. Whereas the pure placebo is an inert substance with no pharmacological effect, the impure placebo is an active substance, which has no medicinal benefit as treatment for the condition it targets, such as vitamins or antibiotics in viral infections [3, 4]. Placebo and its specific effect became a subject of interest and research in itself after Henry Beecher reported on the randomized controlled trial design with a placebo arm during World War II [5, 6].

In this review, we want to give a brief overview about the history of placebo and its effects and its counterpart, nocebo.

## History

The term placebo came from a mistranslation from St. Jerome [7] while he translated the ninth verse in Psalm 116 from Hebrew into Latin. The word ethalecth became placebo, which would mean to please in the English translation [8]. Already long before in ancient times, physicians, priests and magicians used faith healing, embalming, “specific” remedies without pharmacologic effective ingredients and empathy to care for their patients. Today, we know that most of these treatment strategies contain no active substance and, therefore, can be considered as placebo [9].

The Catholic Church, around the sixteenth century, recognized placebo in order to separate imagination from reality by sham procedures. They were trying to filter incorrect exorcisms and therefore used false holy objects for obsessed people to see their reactions. If their responses were like those with real objects, they determined that it was only in their imagination [10].

It took until the eighteenth century for the first controlled clinical studies to be reported. For example, James Lind demonstrated that scurvy could be prevented and even cured trough regular consumption of oranges [8]. The desire arose from such experiments to evaluate medical drugs or procedures by assessing their medical effect to ensure that they could benefit patients [9].

During all this time, the word “placebo” was still used in the Catholic Church. It was associated with fakery, such as “Singers of placebo”. These were paid mourners or mourners who grieved in the hope for a place at the funeral meal. [11] The first doctor, who used placebo in a medical context, was Alexander Sutherland; he called it “fashionable physician placebo” [12].

Another important physician of this time, William Cullen (1710–1790), used placebo to comfort and please patients, whenever he was not sure of his technical ability or knowledge to help them. In these cases, he utilized what we call active/impure placebos. In this content the definition of placebo was not the actual substance, but his intention for the prescription itself [13].

Placebo was first used in a dictionary in 1785, where placebo was defined as a commonplace method of medicine [13]. During time the meaning changed until the 19th century, when placebo received the same definition as today—“an epithet given to any medicine adapted more to please than benefit the patient”. The real placebo effect, however, was not quantified until World War II.

During World War II, Henry Beecher monitored soldiers, who were given plain saline solution when the morphine supply ran out and their pain reaction on the placebo. His observations led to the conclusion that this placebo had an effect of 40% [9].

Today the placebo effect is still a hot scientific research and clinical topic. It is used as a control arm in randomized controlled trials (RCT), to quantitatively distinguish the effect of the active agent from that of placebo in order to determine the true pharmacological effect. The drug has to prove its effectiveness and adverse event profile compared to placebo for approval by regulatory agencies [9]. In many diseases, the ethical basis for using a placebo as control arm is debated.

## How does it work?

The placebo response is due to multiple components, which can be summed up as a psychosocial context or even an atmosphere influencing the therapeutic outcome. These elements may hold an equally high importance as the potency of the drug itself when performing the treatment. The psychological and social aspects of placebo should not only be referred to as the effect of an inactive substance but be seen as words of medical actions capable of impacting the patient’s brain [14].

## Expectation

Most studies identify expectation as one of the main mechanisms for the placebo effect. In neuroscience, the placebo response represents a helpful tool for the understanding of the mechanisms underlying higher brain function, particularly including the reward system [14].

When a patient has a positive expectation for a treatment, there is a higher likelihood of a response through the decrease of self-defeating thoughts and a possible activation from the reward circuit. The potential benefit for the patient’s health and life is a big motivator for the expectations. A key role in this reward circuit is the nucleus accumbens. Indeed, various studies showed an increased activity in this area using imaging modalities [14]. The dopaminergic and opioid activity are increased in the nucleus accumbens in patients with a high placebo response [14].

Different forms of treatments correlate with different expectations from the patient.

A higher dose of pills, a higher price, a well-known brand label and the advertisement lead to higher efficiency, potency and trust in the drug. There are also studies investigating the impact of the color of the drug on the placebo effect. Warm colors contribute to excitement and stimulation, whereas cold colors lead to a feeling of calmness and relaxation. Invasive methods create higher expectations towards a positive treatment outcome and thus result in a larger placebo effect [14].

## Classical conditioning

Classical conditioning also has a significant impact on the placebo effect. Patients generally associate drug therapy with getting better health wise. Doctors, stethoscopes, white coats and hospitals are strong medical symbols. A special kind of conditioning is social learning, where you learn by watching and imitating other people. Therefore people who respond well to placebo, can stimulate others to experience the same [14].

## Doctor-patient relationship

Personal beliefs and memories, verbal information by mental health care providers, treatment characteristics, doctor-patient relationship, interaction with other people in the surrounding and the healthcare setting itself are all part of the context and backbone for the placebo effect. The doctor-patient relationship in particular is an essential ingredient for a strong placebo effect [14].

From the patient’s perspective, the meeting with the doctor is associated with hope, trust and expectations towards an improvement. Meeting the doctor itself represents a reward, as the patient expects a positive outcome. To have trust in another person is linked to the hormone oxytocin, which plays an important role when it comes to social interactions and cognitions. Untrustworthiness, on the other hand, results from the activation of the amygdala. Untrustworthy judgments can be blocked by the binding of oxytocin to its receptors in the amygdala [14].

## Nocebo effect

When looking at the definitions of the terms placebo signifying “I will please” and nocebo meaning “I will harm”, it is easily noticeably that these phenomena are opposites. The nocebo effect correlates with a negative context, negative expectations often arising from information about possible adverse effects [16]. This mechanism can be also shown with active drugs through nonspecific adverse effects, which occur during treatment, but cannot be linked with the pharmacological aspect or the dose of the drug [16]. Patient’s perceptions about the safety of a drug [17] and how well their outcome with the drug will be, vary widely [18].

There are different forms of nocebo—a specific form created through a particular negative expectation, which lead to the same outcome or a generic form, where the patient has only vague thoughts and the outcome may be different to the expectations [15]. A possible negative outcome could also mitigate the effect of and/or tolerance to the drug [19].

The nocebo effect can be observed in different societies, in different ways and intensities. An example are some Latin American and African tribes, where voodoo and witch power are commonly practiced and known. Also, in the western world, information about possible negative side effects can create negative expectations and imaginations resulting in negative events. From a neuroscientific point of view, nocebos can be seen as stressors leading to anticipatory anxiety [14]. The neurobiological mechanism of action of the nocebo effect is not well clarified due to ethical difficulty to design a study [14, 20].

## Discussion

To fully understand the placebo effect and its mechanisms, more research is needed. Not only do we lack reproducible and valid proof of placebo’s real potential, it is also difficult to separate the true placebo effect from cofactors, which have the potential to modulate the response.

Without a no treatment group, we would not be able to assess whether diseases are self-limiting without any treatment. Another cofactor would be the regression towards the mean, which occurs in chronically ill patients. Their symptoms vary and they visit the doctor most times during the peak of the symptoms, followed by a usual reduction over time [21].

There is also the bias that the patient tells the doctor what he thinks that the doctor would want to hear [22]. Another factor is the Hawthorne effect that influences symptoms by the fact that patients are part of a clinical trial [23].

Central to the ethical question regarding placebo is deception. Doctors should value the trust of the patients in them, but health care providers can use our knowledge of placebo mechanism to reduce the patient’s anxieties and improve positive expectations. Through enhancing the psychosocial context and a supportive clinical environment with a good patient-doctor relationship, physicians can garner the placebo effect to the patient’s benefit [24].

The reverse to placebo is nocebo, which remains heavily under-investigated and misunderstood. The drug leaflet often sets the stage for negative effects [15].

## Conclusion

The usage of placebo was widely recognized and used in ancient times. Societies from all around the world have always described specific forms of innocuous treatments resulting in positive changes, performed in highly varied backgrounds and scenarios. [10].

The placebo response itself comprises a wide range of diverse components coming all together to create a positive outcome to alleviate the patient’s suffering. Not just the administered drug but the whole setting around the patient is responsible for creating a beneficial therapeutic effect. It is especially necessary to turn one’s attention to the mind-body interaction as our brain holds a tremendous power over physiological processes [14]. The surroundings, in which the medical procedure is being carried out, has an immense impact on the terminal outcome, no matter if it turns out to be positive or negative.

Through experience, conditioning and learning, placebo effects often occur unconsciously but highly frequently [25]. The mere act of consulting a doctor when suffering, contributes to the placebo response as the doctor is associated with a healer’s position, who will improve the patient’s discomfort [26]. The placebo effect is not only real but also significant. It remains, however, poorly investigated, rarely understood and insufficiently used.
